# Multi-omics research on common allergens during the ripening of pollen and poplar flocs of *Populus deltoides*

**DOI:** 10.3389/fpls.2023.1136613

**Published:** 2023-06-16

**Authors:** Wei Guo, Hui Luo, Yi Cao, Ziyun Jiang, Hui Liu, Jie Zou, Changle Sheng, Yilong Xi

**Affiliations:** ^1^School of Ecology and Environment, Anhui Normal University, Wuhu, China; ^2^School of Basic Medicine, Wannan Medical College, Wuhu, China

**Keywords:** proteomics, metabolomics, pollen, poplar flocs, *Populus deltoides*

## Abstract

**Background:**

*Populus deltoides* is widely cultivated in China and produces a large number of pollen and poplar flocs from March to June per year. Previous studies have found that the pollen of *P. deltoides* contains allergens. However, studies on the ripening mechanism of pollen/poplar flocs and their common allergens are very limited.

**Methods:**

Proteomics and metabolomics were used to study the changes of proteins and metabolites in pollen and poplar flocs of *P. deltoides* at different developmental stages. Allergenonline database was used to identify common allergens in pollen and poplar flocs at different developmental stages. Western blot (WB) was used to detect the biological activity of common allergens between mature pollen and poplar flocs.

**Results:**

In total, 1400 differently expressed proteins (DEPs) and 459 different metabolites (DMs) were identified from pollen and poplar flocs at different developmental stages. KEGG enrichment analysis showed that DEPs in pollen and poplar flocs were significantly enriched in ribosome and oxidative phosphorylation signaling pathways. The DMs in pollen are mainly involved in aminoacyl-tRNA biosynthesis and arginine biosynthesis, while the DMs in poplar flocs are mainly involved in glyoxylate and dicarboxylate metabolism. Additionally, 72 common allergens were identified in pollen and poplar flocs at different developmental stages. WB showed that there were distinct binding bands between 70 and 17KD at the two groups of allergens.

**Conclusions:**

A multitude of proteins and metabolites are closely related to the ripening of pollen and poplar flocs of *Populus deltoides*, and they contain common allergens between mature pollen and poplar flocs.

## 1 Introduction

Allergic diseases (such as allergic asthma, hay fever) are mainly chronic inflammatory diseases involving a variety of inflammatory cells mediated by IgE (Yip et al., 2016; Helou et al., 2020). The incidence and prevalence of allergy is increasing year after year in the past few decades, which has become issues of public health concern (Wesemann and Nagler, 2016; Liu et al., 2020). Among the inhalant allergens, the pollen is one of the most important sources responsible for eliciting allergic symptoms. Studies have shown that Bet v 1, the major allergen in birch pollen, causes allergic symptoms in more than 20% of the European population, and 70% of allergic patients to birch pollen exhibit different degrees of cross-reaction on food (Erler et al., 2011; Negi and Braun, 2017). In northern China, Art v 1 from *Artemisia* pollen is a major allergen causing allergic diseases, and more than 50% of patients with respiratory allergies are allergic to this pollen (Gao et al., 2019). The study of local pollens and allergens is of great significance for the prevention and immunotherapy of allergic diseases.

*Populus deltoides* originates from North America, which has important ecological and economic value due to its fast growth and good disease resistance (Bai et al., 2021). *P. deltoides* is particularly suitable for riparian habitats and plays an important role in the early and late riparian niches; in addition, *P. deltoides* can increase the proportion of water absorbed by shallow soil, which is of great significance for inhibiting frequent extreme precipitation and flood events (Shakya et al., 2013; Zhang et al., 2020). Hence, *P. deltoides* is widely cultivated in the Middle-Lower Yangtze Region in China to protect levees, and produces a large number of pollen and seeds with pappus (commonly known: poplar flocs) every year. Previous studies have found that the pollen of *P. deltoides* contains a large number of allergens, and can induce allergic asthma (Guo et al., 2021). In China, *P. deltoides* blooms (male) in March and produces poplar flocs (female) in May. Assuming that pollen and poplar flocs contain common allergens, it’s equivalent to prolonging allergen exposure. However, at this stage, there is very limited research about whether the pollen and poplar flocs of *P. deltoides* contain common allergens, and the characteristics of allergen expression levels in pollen and poplar flocs at different developmental stages. In addition, it has not been reported that the common allergens in the pollen and poplar flocs are prone to cross-allergic reactions with others.

The contents of proteins vary greatly and play an important role in plant growth. Metabolites can not only reflect the phenotypic state of the organism, but also participate in regulating protein interactions as functional regulatory substances, affecting protease activity and protein stability, and regulate the metabolic state of the organism through a feedback mechanism. Proteomics and metabolomics are powerful tools for studying protein and metabolite components in plant organs. Identification of allergens and related metabolites in pollen and fruit by multi-omics has become a heated topic in current research (Salzano et al., 2019; Tao et al., 2022). In this study, proteomics and metabolomics were used to identify the common allergens in the pollen and poplar flocs of *P. deltoides*; in addition, their expression characteristics and related metabolites at different developmental stages were analyzed. This study not only helps reveal the molecular mechanism of pollen and poplar flocs maturation, but also provides a theoretical support for the prevention of allergic diseases.

## 2 Methods

### 2.1 Collection of samples

**Figure 1:**
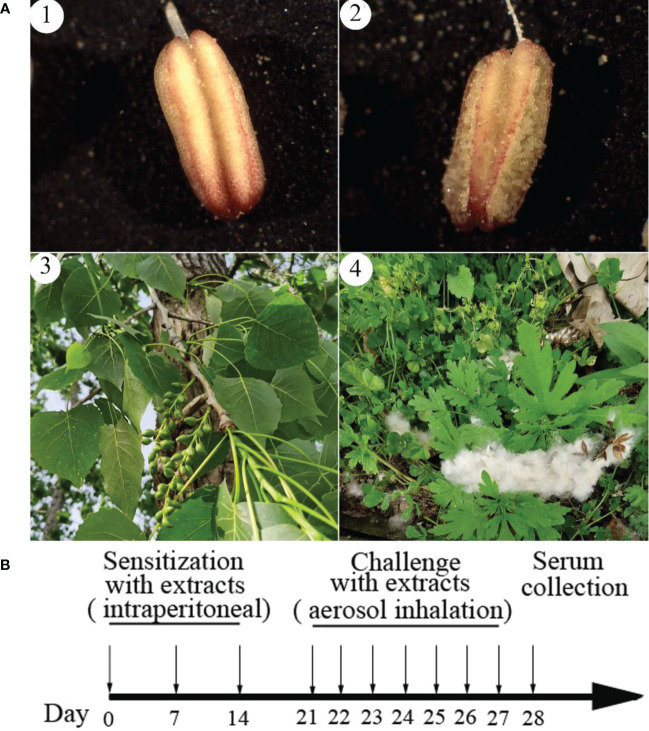
The pollen and poplar floc of *Populus deltoides* at different developmental stages and the calendarization of mice immunization with their extracts. Poplar floc is seeds with pappus. **(A)**, 1: Immature anthers containing immature pollen; 2: Mature anthers and mature pollen; 3: Fruit growing on *Populus deltoides* containing unripe poplar floc; 4: The fruit is ripe, it falls off the tree and the poplar floc fly out. **(B)**, the calendarization of mice immunization with extracts from pollen and poplar flocs.

*P. deltoides* pollen and poplar flocs samples used in this study were collected from March to June 2022 in the Wuhu section of the Yangtze River levee (N31.33, E118.38), different trees with the same height and direction). According to the laws of phenology, 7 days after inflorescence protrudes, pollen in indehiscent anthers is defined as immature pollen; the scattered pollen after dehiscent anthers is defined as mature pollen; 20 days after fruit set, poplar flocs in indehiscent pericarp are defined as immature poplar flocs; poplar flocs in dehiscent pericarp are defined as mature poplar flocs (**Figure 1A**). Collection of immature pollen: Tree pruner cuts off the branches, collects the anthers, brings them back to the laboratory immediately, uses a sharp knife blade to cut the anthers under a microscope, and picks out the pollen with an anatomical needle. Collection of mature pollen: The sampling belt encloses the inflorescence and shakes it violently to shed the pollen. All samples shall be stored in -80°C refrigerator immediately after purification.

### 2.2 Proteomic profiling

Lab-free quantitative proteomics was performed with reference to previous study (Guo et al., 2021). The main processes were as follows: Weigh the appropriate sample into the mortar, add liquid nitrogen and grind thoroughly. The 800μL extraction buffer [Sucrose 2.4 g, NaCl 0.058 g, EDTA·2Na 0.146 g, DTT 0.02 g, 0.5M Tris-HCl (pH6.8) 2.5 mL, 1.5M Tris-HCl (pH8.8) 2.5mL, add ddH_2_O to 10mL, dissolve and mix thoroughly] was added to each sample and the mixtures were added with equal volume of Tris-phenol buffer (6.8M, Solarbio life sciences, Beijing, China) and mixed for 30min at 4°C. Then, the mixtures were centrifuged at 7000g for 10min at 4°C to collect phenol supernatants. The supernatants were added for 5 times the volumes of ammonium acetate-methanol buffer (0.1M, Sangon, Shanghai, China) and precipitated at -20°C overnight. Later, the samples were centrifuged at 12000g for 10min to collect precipitation and washing it with cold methanol (A456-4, ThermoScientific, Waltham, USA). The precipitation was collected by repeated centrifugation and methanol was replaced with acetone (LOT NO. 40064485, OKA, Shanghai, China). The samples were centrifuged at 12000 g for 10 min at 4°C to collect precipitation and the precipitation were dried at room temperature and dissolved in SDS lysis buffer (LOT NO. P0013G, Beyotime, Shanghai, China). Finally, the samples were centrifuged at 12000g for 10min to collect supernatants. The supernatant is the total protein of the sample. Then, the protein concentration was detected by BCA Kits (LOT NO. 23225, ThermoScientific, Waltham, USA). Protein quality was determined by 12% SDS-PAGE and digested with 0.1% trypsin. Then, the peptide was labeled with TMTpro reagent (LOT NO. A44521, ThermoScientific, Waltham, USA). All analyses were performed by a QE mass spectrometer (MS) (ThermoFisher, USA) equipped with an Easyspray source, the samples were sent to the precolumn Acclaim PepMap100 100 μm×2 cm (RP-C18, Thermo Fisher) at a flow rate of 300 nL/min and separated by analytical column Acclaim PepMap RSLC, 75μm×50cm (RP-C18, Thermo Fisher). The first-order MS mass resolution was set to 60000, the automatic gain control value was set to 3e6, the maximum injection time was set to 50 ms, the MS scanning was set to the full scanning charge/mass ratio (m/z) range of 350-1500, and 20 of the highest peaks were scanned by MS/MS. Proteome Discover 2.4 (Thermo Fisher) was used for database retrieval.

### 2.3 Identification of potential allergens

The amino acid sequences were retrieved from the Uniprot database according to the protein accession provided by proteomics (https://www.uniprot.org/). In the AllergenOnline database (http://www.allergenonline.org/), full-length sequences were retrieved by FASTA alignments, matching with known allergens, more than 50% were identified as potential allergens, and indicates the possibility of cross-reaction between them; more than 70% is considered to be susceptible to cross-reaction between them (Aalberse, 2000; Abdelmoteleb et al., 2021).

### 2.4 Metabolome analysis

Sample preparation: 40 mg samples (4 replicates) were weighed into eppendorf tubes, 20 μL of L-2-chlorophenylalanine (LOT NO. C2001, HC Biotech, Shanghai, China) as internal standard (0.06 mg/mL, dissolved in methanol) and 1 mL mixture of methanol-water (vol/vol = 7/3) were added to each sample. The mixture was ultrasonic in ice bath for 30 minutes and rested overnight at -20°C. After centrifugation for 10 min (13000 rpm, 4°C), 150 μL supernatant was absorbed and filtered by 0.22 μm organic phase pinhole filter, and transferred to LC sample vial for LC-MS analysis. ACQUITY ultra performance liquid chromatography (UPLC) I-Class system (Waters, USA) coupled with VION IMS QTOF MS (Waters, USA) was used to analyze the metabolic profiling in both ESI positive and ESI negative ion modes. An ACQUITY UPLC BEH C18 column (1.7 μm, 2.1 × 100 mm) were used in both positive and negative modes. Water and Acetonitrile (LOT NO. A998-4, ThermoScientific, Waltham, USA)/Methanol 2/3 (v/v), both containing 0.1% formic acid were used as mobile phases A and B, respectively. The flow rate was 0.4 mL/min at 45°C of column temperature. All the samples were kept at 4°C during the analysis. The injection volume was 1 μL. Finally, data acquisition was performed in full scan mode (m/z ranges from 50 to 1000) combined with MSE mode and data were analyzed with reference to relevant literature (Liu et al., 2019).

### 2.5 Data preprocessing

As for proteomics: The LC-MS/MS raw data were imported in Maxquant for the analysis of labeling free quantification. Target-decoy-based error discovery rate (FDR) method is used to limit random peaks. The mass and strength of peptide peaks in mass spectrometry (MS) spectra were detected to identify peptides and assembled into three-dimensional (3D) peak hills over the m/z retention time plane, which are filtered by applying graph theory algorithms to identify isotope patterns. To achieve high mass accuracy, weighted averaging and mass recalibration (subtracting the determined systematic mass error from the measured mass of each MS isotope pattern) were conducted. Peptide and fragment masses were searched in an organism specific sequence database (including the target sequences, reverse counterparts and contaminants). About metabolomics: The original LC-MS data were processed by software Progenesis QI V2.3 (Nonlinear, Dynamics, Newcastle, UK) for baseline filtering, peak identification, integral, retention time correction, peak alignment, and normalization. Compound identification were based on precise mass-to-charge ratio (M/z), secondary fragments, and isotopic distribution using The PlantMADS Database (PMDB). The extracted data were further purified by removing any peaks with a missing value in more than 50% in groups, by replacing 0 value by half of the minimum value, and by screening according to the qualitative results of the compound (>36). A data matrix was combined from the positive and negative ion data. A combination of multidimensional analysis and one-dimensional analysis was used to screen different metabolites between groups. In OPLS-DA analysis, variable important in projection (VIP) can be used to measure the influence of the expression patterns of metabolites on the classification and discrimination of samples of each group, so as to explore the differential metabolites of biological significance. *T* test was further used to verify the significance of different metabolites between groups.

### 2.6 Preparation of specific antibodies

The mature pollen or poplar flocs was grinded in liquid nitrogen and extracted with phosphate buffer saline (PBS, 0.2M, pH 7.5) according to the ratio of solid to liquid 1:20 (g:mL, 4°C, 48h). The extract was filtered by filter paper to remove the residue and sterilized by a 0.22μm filter. The concentration of the extract was detected by BCA Kits. Thirty specific pathogen free (SPF) BALB/c mice were randomly divided into 3 groups (n = 10, license number: SCXK 2020-0005), which were PBS group, pollen group and poplar flocs group. Sensitized mice in pollen group and poplar flocs group were intraperitoneally injected with 10 µg total protein of pollen or poplar flocs extract [contains 4% Al(OH)_3_] on day 0, 7, and 14, respectively; On the 21st day, pollen group and poplar flocs group were stimulated by 0.5 μg/mL pollen or poplar flocs extract for challenge, 30 min/time for one week; PBS groups were treated with equivalent volumes of PBS; 24h after the last stimulation, blood was taken from the eye socket in each group and placed it in a centrifuge tube (**Figure 1B**). After standing at 4°C for 2h, the serum was collected by centrifugation at 4000 g for 5 mins.

### 2.7 Western blot

The plant protein extraction kit (LOT NO. C500053, Sangon,Shanghai, China) was used to extract the total protein of mature pollen/poplar flocs and protein concentration was detected by BCA Kits; 12% SDS-PAGE for 30 µg protein; the total protein was transferred to the nitrocellulose membrane and wash it 3 times with 1×tris-buffered saline with 0.1% tween 20 (TBST) [10×TBST (0.1 M Tris, 1.5 M NaCl, 1% Tween-20, pH7.5, LOT NO. C520009-0500, Sangon, Shanghai, China) was diluted with deionized water]. Then, the membrane was blocked with 5% non-fat milk in TBST for 2 h and wash it 3 times with 1×TBST. Afterward, the total protein of pollen was incubated with primary antibody (sensitized serum with poplar flocs), the total protein of poplar flocs was incubated with primary antibody (sensitized serum with pollen, diluted at 1:1000) at 4°C overnight, followed by washing 3 times with 1×TBST, HRP-labeled rabbit anti-mouse IgG (LOT NO. 6170-05 Amyjet, Wuhan, China) as secondary antibody (diluted at 1:1000), Incubated at room temperature for 2h.Wash the film with 1×TBST for 3 times, prepare the developing liquid A and B at 1:1 (LOT NO. BL520A, Biosharp, Guangzhou, China), and the film was exposed immediately on the Image System (ChemiDoc MP, Bio-RAD).

## 3 Results

### 3.1 Proteomic characterization of pollen and poplar flocs in *P. deltoides*

**Figure 2:**
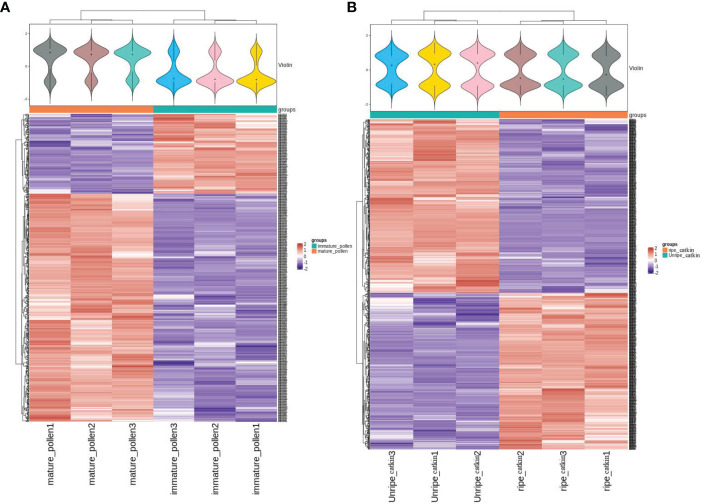
Expression pattern clustering heat map. **(A)**, Clustering heat map of pollen expression patterns at different developmental stages. Above: probability distribution of expression values reflected by the contour of violin case above, different color filling represents different samples, “+” in the middle of violin represents the median of sample expression quantity, each sample has three repetitions, and the median is on the same level, indicating good repeatability of the sample. Below: The clustering heat map is classified according to protein expression level, red represents high-expression protein, blue represents low-expression protein, and each line represents the expression level of protein in different groups. **(B)**, Clustering heat maps of expression patterns of poplar flocs at different developmental stages.

A total of 3914 identical proteins were identified from pollen and poplar flocs at different developmental stages (**Supplementary 1**); 564 differentially expressed proteins (DEPs) were involved in pollen maturation, among which 418 were up-regulated (multiprotein-bridging factor 1 MBF1, such as A0A2I4GFN0; NOI4-like protein, such as A0A2I4GL39; Cysteine proteinase inhibitor, such as A0A2I4EGF0), 146 down-regulated proteins(18KD heat shock protein, such as A0A2I4FG07 and A0A2I4FFZ4; 40S ribosomal protein, such as A0A2I4GHC8; Glucan synthase, such as A0A2I4F0F4). A total of 836 DEPs were involved in poplar flocs maturation, among which 397 were up-regulated (legumin B-like, such as A0A2I4GEI2; 11S globulin mainly, such as A0A1L6K371), and 439 were down-regulated(endoglucanase, such as A0A2I4GR72; GDSL esterase, such as A0A2I4EEB5). The expression trend of DEPs in pollen and poplar flocs at different developmental stages was stable (**Figure 2**).

### 3.2 Functional enrichment analysis of DEPs

**Figure 3:**
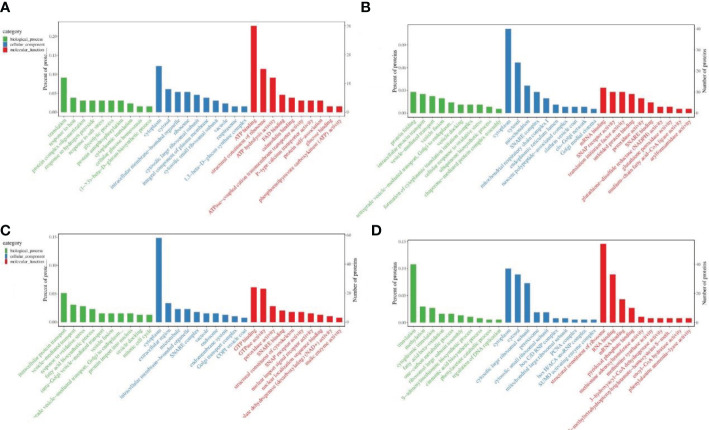
GO enrichment analysis of DEPs in pollen and poplar flocs at different developmental stages. **(A, B)** GO enrichment analysis of down/up-regulated proteins during pollen maturation. **(C, D)** GO enrichment analysis of down/up-regulated proteins during poplar flocs maturation. X axis, the entry name of the GO enrichment; Y axis left, the percentage of proteins in the corresponding entry; Y axis right, the number of proteins in the corresponding entry.

Biological functions of DEPs were described by GO enrichment analysis. During pollen maturation, down-regulated proteins were mainly enriched in translation, response to heat, cytoplasm, ATP binding, structural constituent of ribosome, and ATP hydrolysis activity in terms of biological processes, cell components and biological functions (**Figure 3A**); the up-regulated proteins are mainly enriched in protein folding, intracellular protein transport, cytoplasm, mRNA binding, and translation initiation factor activity (**Figure 3B**). During poplar flocs maturation, down-regulated proteins were mainly enriched in protein transport, response to oxidative stress, cytoplasm, GTP binding, GTPase and peroxidase activity (**Figure 3C**); the up-regulated proteins are mainly enriched in translation, methylation, cytoplasm, RNA, mRNA and pyridoxal phosphate binding (**Figure 3D**).

**Figure 4:**
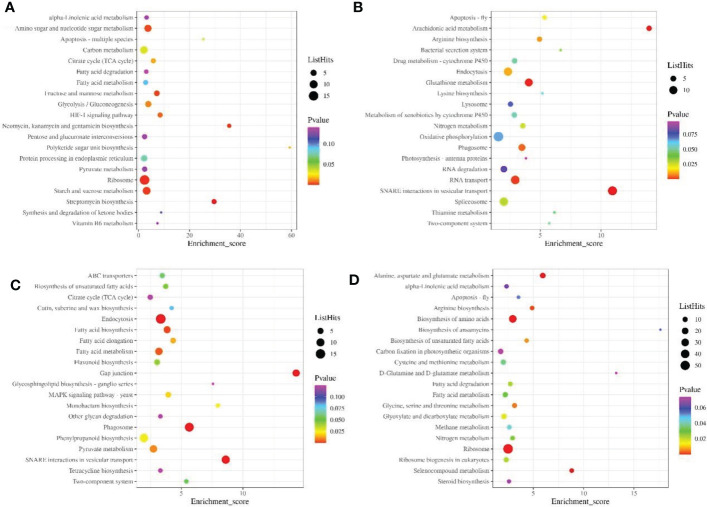
KEGG enrichment analysis of DEPs in pollen and poplar flocs at different developmental stages. **(A, B)** KEGG enrichment analysis of down/up-regulated proteins during pollen maturation. **(C, D)** KEGG enrichment analysis of down/up-regulated proteins during poplar flocs maturation. X axis, enrichment Score; Y axis, the top20 pathway; the larger the bubbles, the higher the number of DEPs.

KEGG enrichment analysis showed that the down-regulated proteins were mainly involved in ribosome, carbon, starch and sucrose metabolism during pollen maturation (**Figure 4A**), up-regulated proteins are mainly involved in Oxidative phosphorylation, Spliceosome, Endocytosis and other signaling pathways (**Figure 4B**). During poplar flocs maturation, down-regulated proteins are mainly involved in phenylpropanoid biosynthesis, endocytosis and pyruvate metabolism (**Figure 4C**), up-regulated proteins are mainly involved in signaling pathways such as ribosome, biosynthesis of amino acids, glyoxylate and dicarboxylate metabolism (**Figure 4D**).

### 3.3 Metabolite profiles of pollen and poplar flocs at different ripening periods

**Figure 5:**
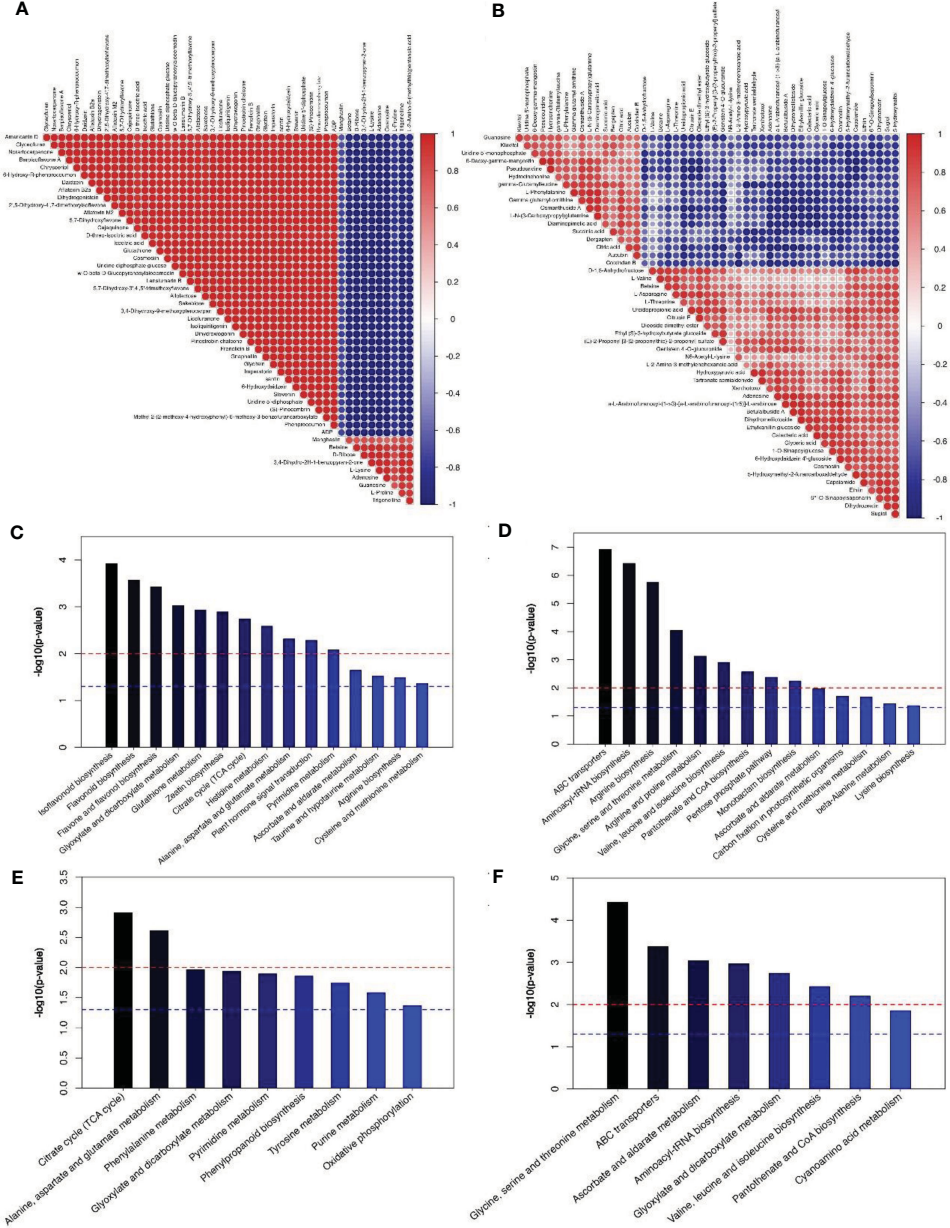
Correlation and KEGG enrichment analysis of Top50 metabolites during pollen and poplar flocs. **(A, B)** Red means positive correlation, blue means negative correlation; the larger the dot, the greater the correlation coefficient. **(C–F)** The red line, *P* < 0.01, blue line, *P* < 0.05; when the top of the bar is higher than the blue line, the signal path is significant.

According to metabolomics analysis, a total of 354 differential metabolites (DMs) were identified in the pollen group, which were mainly prenol lipids (15.32%) and flavonoids (14.81%). During pollen maturation, 249 DMs were up-regulated (such as albafuran C, gomphrenin II and sinensetin etc.) and 105 were down-regulated (such as dehydroanonaine, licoagroaurone and hydroxymyricanone etc.), among which the up-regulated DMs such as gnaphaliin, isoliquiritigenin, etc. and the down-regulated DMs such as Adenosine, D-Ribose etc. were considered to significantly affect pollen maturation (**Supplementary 2**). Moreover, 105 DMs have been identified during poplar flocs maturation, of which 42 are up-regulated (such as 8-Hydroxy-4’-methoxypinoresinol and capsianoside I etc.) and 63 are down-regulated (such as S-Allylcysteine and genistein 4’-O-glucuronide etc.), which main compounds are prenol lipids (14.49%) and organooxygen compounds (13.99%) (**Supplementary 3**). In pollen group, there was a significant correlation between different metabolites (*P* < 0.05, **Figure 5A**), as well as poplar flocs group (**Figure 5B**).

KEGG enrichment analysis showed that up-regulation of DMs in pollen group mainly involves in flavone and glutathione biosynthesis (**Figure 5C**); the down-regulation of DMs mainly involves in aminoacyl-tRNA biosynthesis, arginine biosynthesis (**Figure 5D**). Up-regulation of DMs in poplar flocs group mainly involved in citrate cycle, glutamate and phenylalanine metabolism (**Figure 5E**); the down-regulation of DMs mainly involves glycine, serine and threonine metabolism (**Figure 5F**).

### 3.4 Components and expression characteristics of potential allergens

According to sequence alignment in AllergenOnline database, 72 potential allergens from more than 10 protein families were identified. The amino acid sequences of potential allergens are more than 50% similarity to those of known allergens reported in the AllergenOnline database. Except for 11 allergens such as A0A2I4GA71, the expression levels of others were significantly different in different developmental stages of pollen and poplar flocs (**Table 1**).

**Table 1:** Potential allergens in pollen and poplar flocs of *P. deltoides* and their expression characteristics at different developmental stages.

Allergenic protein family	Protein accession	Similarity (%)	Protein description	Relative Abundance		
				immature pollen/mature pollen	mature pollen/unripe fruit	unripe fruit/ripe fruit
Calcium-binding	A0A2I4EH85	Bet v 3 (82.3%)	Calcium-binding allergen Bet v 3 OS=Juglans regia	0.68	10.88	NS
	A0A2I4E3I2	Cyn d 7 (66.7%)	Calcium-binding protein CML21 OS=Juglans regia	NS	7.70	NS
	A0A2I4G3C1	Phl p 7 (82.8%)	Calcium-binding protein CML16 OS=Juglans regia	NS	1.57	1.27
	A0A2I4F3C0	Amb a 5 (57.6%)	Calcium-binding protein 39 OS=Juglans regia	NS	0.56	NS
	A0A2I4GV50	Bra r 2 (76.9%)	Calcium-binding protein CML13 OS=Juglans regia	NS	0.60	1.45
Expansins proteins	A0A833UVW9	Sor h 1 (60%)	Expansin-like EG45 domain-containing protein OS=Juglans regia	0.79	NS	NS
	A0A6P9EYI7	Zea m 1 (65.8%)	Expansin OS=Juglans regia OS=Juglans regia	NS	0.23	1.62
	A0A2I4DR18	Ant o 1 (72%)	Expansin OS=Juglans regia	NS	0.19	1.77
Heat shock protein 70	A0A2I4DW30	Mala s 10 (68.9%)	Heat shock 70 kDa protein 16-like	NS	1.54	NS
	A0A2I4FTU2	Pen c 19 (67.4%)	Heat shock 70 kDa protein 17	NS	1.47	NS
	A0A2I4H3J1	Mala s 10 (65.7%)	Heat shock 70 kDa protein 16- like isoform	NS	1.42	NS
	A0A2I4GA71	Mala s 10 (67.9%)	Heat shock 70 kDa protein 15-like	**NS**	**NS**	**NS**
	A0A2I4DXX6	Mala s 10 (68.6%)	Heat shock 70 kDa protein 15-like	**NS**	**NS**	**NS**
	A0A2I4E207	Der p 28 (77.7%)	Heat shock 70 kDa protein, mitochondrial-like	NS	1.72	NS
	A0A2I4DLR7	Pen c 19 (89.6%)	Heat shock 70 kDa protein	**NS**	**NS**	**NS**
	A0A2I4GQV9	Der p 28 (77.6%)	Heat shock 70 kDa protein, mitochondrial-like	NS	1.37	NS
	A0A2I4EV32	Tyr p 28 (89.6%)	Heat shock 70 kDa protein 4-like	**NS**	**NS**	**NS**
	A0A2I4F5E4	Aed a 8 (79%)	Heat shock 70 kDa protein, mitochondrial	**NS**	**NS**	**NS**
Profilins	A0A2I4DNN6	Jug r 7 (100%)	Profilin OS=Juglans regia	NS	5.27	NS
	A0A2I4HKI3	Cor a 2 (98.5%)	Profilin OS=Juglans regia	NS	1.85	NS
	A0A2I4GR27	Hev b 8 (98.5%)	Profilin OS=Juglans regia	**NS**	**NS**	**NS**
Peroxidases	A0A2I4HQ39	Lat c 6 (50.0%)	L-ascorbate peroxidase	**NS**	**NS**	**NS**
	A0A2I4H8K8	Alt a 10 (64.4%)	Glutathione peroxidase	0.69	NS	NS
	A0A2I4DHB7	Chi t 4 (64.3%)	Glutathione peroxidase	0.79	0.63	1.30
	A0A2I4DLU5	Pan h 1 (65.4%)	Peroxidase OS=Juglans regia	NS	0.22	2.02
	A0A2I4GG94	Art v 5 (56.4%)	Peroxidase OS=Juglans regia	NS	0.16	1.23
	A0A833XKN9	Pyr c 1 (60%)	Peroxidase OS=Juglans regia	NS	0.14	1.44
Polygalactur-onase	A0A2I4H1N9	Pla a 2 (83%)	Exopolygalacturonase-like	NS	17.19	NS
Proteases	A0A2I4ENU0	Cuc m 1 (70.7%)	Subtilisin-like protease SBT3.17	NS	2.59	NS
	A0A6P9EHT5	Pan h 4 (54.5%)	Lon protease homolog 2, peroxisomal	**NS**	**NS**	**NS**
	A0A2I4GL90	Asp f 8 (71.1%)	ATP-dependent zinc metalloprotease FTSH 4, mitochondrial-like	NS	1.28	NS
	A0A2I4F2I5	Par o 1 (66.7%)	Mitochondrial inner membrane protease subunit	**NS**	**NS**	**NS**
	A0A2I4DEK9	Asp o 13 (57.8%)	Subtilisin-like protease SBT6.1 isoform X1	**NS**	**NS**	**NS**
	A0A2I4FTS9	Cuc m 1 (68.6%)	Subtilisin-like protease SBT1.4	1.52	0.30	1.55
	A0A2I4EKJ9	Cuc m 1 (63.7%)	Subtilisin-like protease SBT3	NS	0.42	1.33
	A0A2I4ERI4	Tri a 45 (62.7%)	Serine protease EDA2	NS	0.45	NS
	A0A2I4EFL0	Rhi o 1 (50.9%)	Aspartyl protease AED3-like	NS	0.51	NS
	A0A2I4DKI0	Cuc m 1 (68.5%)	Subtilisin-like protease SBT1.7	NS	0.25	NS
	A0A2I4GS51	Der p 2 (59.3%)	Aspartyl protease family protein 2-like	NS	0.18	1.87
	A0A6P9EI45	Car p 1 (76.7%)	Cysteine protease XCP1-like	NS	0.16	NS
	A0A2I4GZU9	Rhi o 1 (50.1%)	Aspartyl protease AED3-like	NS	0.10	1.54
Patatin	A0A2I4H6Z2	Sola t 1 (73.2%)	Patatin OS=Juglans regia	**NS**	**NS**	**NS**
	A0A2I4HKT5	Hev b 7 (83.1%)	Patatin OS=Juglans regia	NS	0.18	1.33
Proteinase inhibitor	A0A2I4GZP3	Act d 4 (66.7%)	Cysteine proteinase inhibitor	0.41	5.10	1.46
	A0A2I4EGF0	Act d 4 (73.7%)	Cysteine proteinase inhibitor	0.36	7.94	NS
	A0A2I4E6P0	Ole e 5 (67.4%)	Pectinesterase inhibitor 51	NS	0.49	NS
	A0A2I4GID5	Act d 4 (64.7%)	Cysteine proteinase inhibitor	0.67	0.55	NS
	A0A2I4GZL4	Sola t 2 (63.2%)	Kunitz trypsin inhibitor 5-like	NS	0.04	4.82
Globulin	A0A1L6K371	Jug n 4 (100%)	11S globulin OS=Juglans nigra	NS	0.81	0.18
Others	A0A2I4GL39	Asp f 4 (91.3%)	Protein NOI4-like isoform X1	0.29	19.0	NS
	A0A833WXQ5	Gly m 5 (52.5%)	SHSP domain-containing protein	1.57	NS	NS
	A0A2I4F0G5	Amb a 4 (61.4%)	RGG repeats nuclear RNA binding protein A	0.55	3.19	NS
	A0A834D2J8	Mala f 3 (71.9%)	Glutaredoxin-dependent peroxiredoxin	0.66	3.99	NS
	A0A833TEN4	Asp n 14 (56.2%)	Glyco_hydro_3 domain-containing protein	1.52	2.73	NS
	A0A6P9EEH3	Can s 3 (51.1%)	Adenylate kinase	0.41	6.09	NS
	A0A2I4ERL8	Asc s 1 (56.9%)	Peptidyl-prolyl cis-trans isomerase	0.37	3.37	NS
	A0A834CU35	Tri a 17 (73.7%)	Beta-amylase OS=Juglans regia	0.74	2.56	NS
	A0A2I4HL04	Api m 10 (62.5%)	Calreticulin OS=Juglans regia	0.72	3.11	0.80
	A0A2I4FEU6	Par j 1 (55.2%)	NADH dehydrogenase flavoprotein 2	0.79	1.40	NS
	A0A2I4GFN0	Pen c 24 (64%)	Multiprotein-bridging factor 1b	0.22	3.11	NS
	A0A2I4GEI2	Pis v 2 (84.1%)	legumin B-like OS=Juglans regia	NS	NS	0.12
	A0A2I4GR72	Lol p 5 (56.5%)	Endoglucanase OS=Juglans regia	0.79	0.21	5.51
	A0A2I4HMQ2	Der p 33 (72.5%)	Tubulin beta chain	NS	NS	1.47
	A0A2I4G145	Sal k 3 (97.2%)	Homocysteine S-methyltransferase	NS	NS	0.71
	A0A2I4EEB5	Hev b 4 (57.6%)	GDSL esterase/lipase At5g33370-like	NS	0.14	4.94
	A0A2I4EMI1	Der p 10 (59.7%)	Kinesin-like protein OS=Juglans regia	NS	0.16	2.72
	A0A2I4FWH9	Lat c 6 (70.3%)	Ras-related protein RABE1c	NS	NS	1.29
	A0A6P9ENN3	Lat c 6 (75.6%)	Ras-related protein RABB1c	NS	0.80	1.30
	A0A2I4DXM6	Bla g 2 (67.5%)	Aspartic proteinase-like isoform X2	NS	0.47	0.66
	A0A833XCG4	Lup an 1 (74.2%)	Uncharacterized protein OS=Juglans regia	0.80	2.25	0.23
	A0A2I4F1R5	Ole e 11 (59.7%)	Pectinesterase OS=Juglans regia	NS	0.82	0.49
	A0A2I4DE51	Lol p 5 (57.6%)	Phosphomethylpyrimidine synthase	0.71	1.40	0.66

NS, no difference at different developmental stages. Relative abundance < 1, the expression level of the former was lower than that of the latter (P < 0.05); > 1, the expression level of the former was higher than that of the latter (*P* < 0.05). The second column is potential allergens in pollen and poplar flocs of *P. deltoids*; the fourth column is a description of the protein in the Uniprot database.

### 3.5 Co-analysis of proteomics and metabolomics

**Figure 6:**
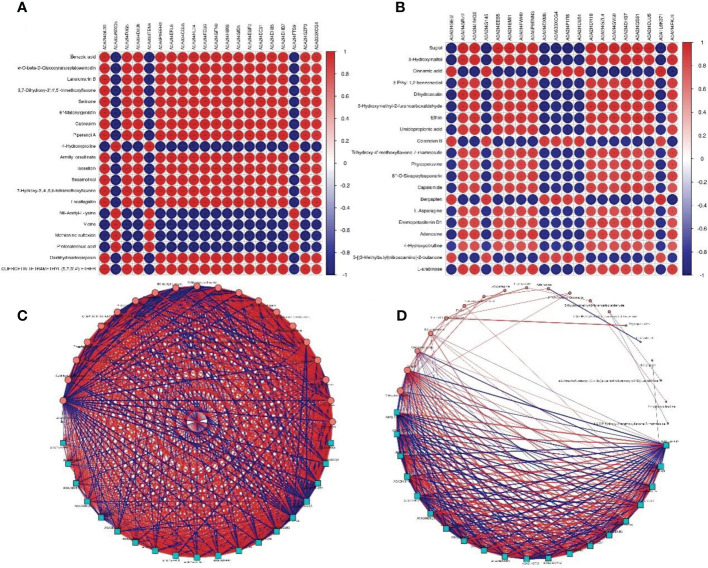
Correlation and interaction analysis between allergens and metabolites. **(A, B)** Row represents a different metabolite, column represents a corresponding protein; red represents a positive correlation, blue represents a negative correlation; the darker the color, the greater the correlation, and the smaller the circle diameter, the smaller the correlation. **(C, D)** Blue squares represent proteins, red circles represent metabolites, the size of the shape represents the interaction frequency, lines between shapes represent the correlation, thickness of lines represents the degree of correlation, red lines represent positive correlation, and blue lines represent negative correlation.

To study the correlation between allergens and metabolites, integrated analysis was performed on the top 20 components in protein abundance and metabolite content. The results showed that there were significant correlations between allergens and metabolites in pollen group (r = -0.9999–0.9998, *P* < 0.01, **Figure 6A**), as well as the poplar flocs group (r = -0.9925–0.99701, *P* < 0.01, **Figure 6B**; **Supplementary 4**). Moreover, significant interactions were found among allergens, metabolites and allergen-metabolites in pollen group (**Figure 6C**) and poplar flocs group (**Figure 6D**; **Supplementary 5**).

### 3.6 Analysis of potential allergens sensitizing components between two groups

**Figure 7:**
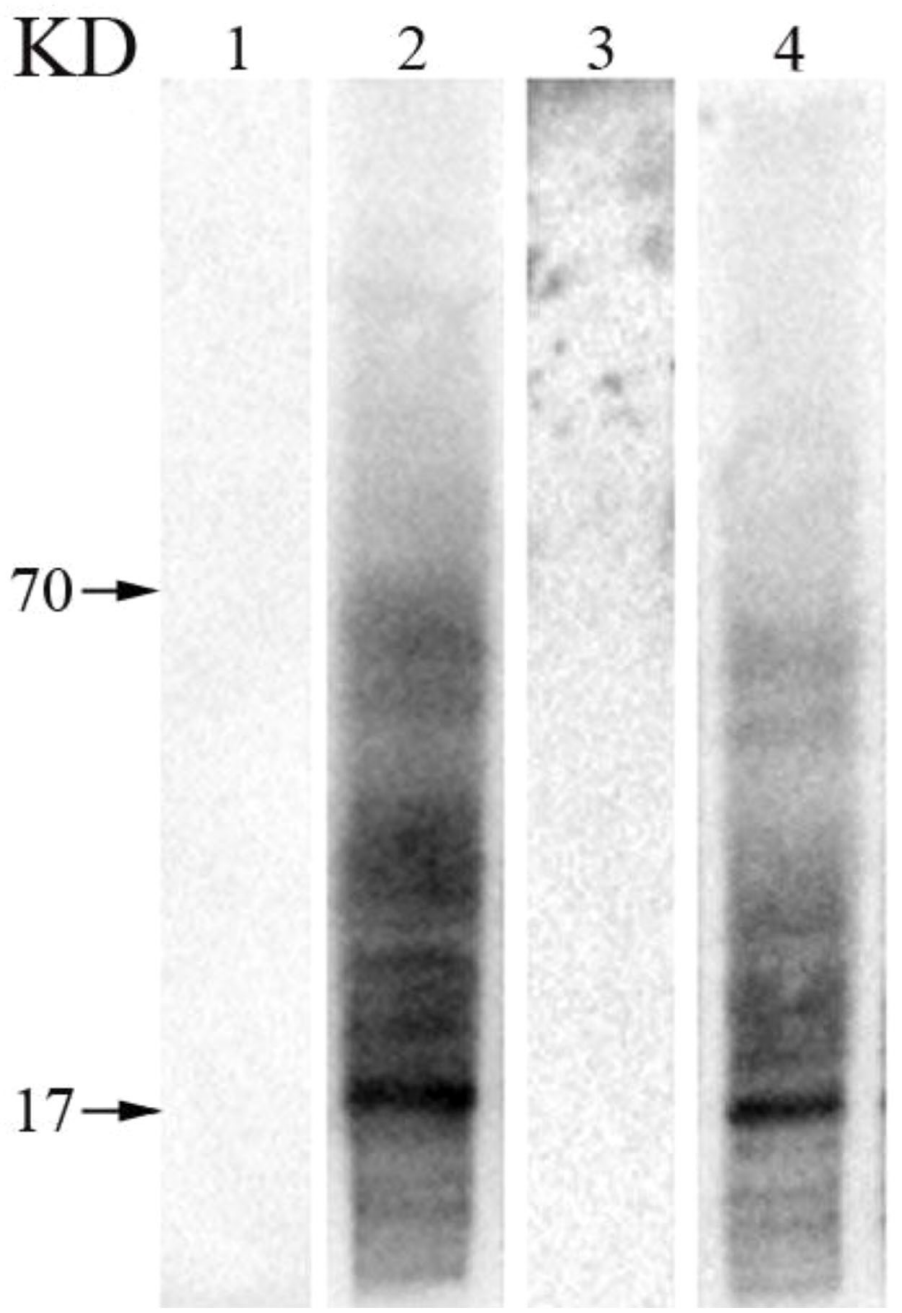
The common allergen components in mature pollen and poplar flocs were verified by WB. 1: Total protein of pollen incubated with PBS sensitizing serum, 2: Total protein of pollen incubated with sensitized serum of poplar flocs, 3: Total protein of poplar flocs incubated with PBS sensitized serum, 4: Total protein of poplar flocs incubated with pollen sensitized serum.

The results of WB showed, no bands were found in total proteins of mature pollen and poplar flocs incubated with PBS sensitized serum. The total protein of mature poplar flocs incubated with sensitized serum showed obvious bands between 70-17KD, and the total protein of mature poplar flocs incubated with sensitized serum also showed the same characteristics (**Figure 7**).

## 4 Discussion

Pollen/fruit ripening is a complex biological process involving physiological, structural and numerous molecular changes (Osorio et al., 2013; Li et al., 2019). In this study, proteomics and metabolomics were used to study the changes of protein and metabolites content during the ripening process in pollen and poplar flocs of *P. deltoides*. Proteomic analysis showed that 18KD heat shock protein (HSP), and 40S ribosomal protein were most significantly down-regulated during pollen maturation. Studies show that male gametophyte (pollen) is very sensitive to heat stress during the whole process of development and maturation, which has an important effect on pollen quantity, morphology, maturation and metabolism (Hedhly, 2011). The variation of sHSPs contents in different subtypes are essential for normal nutrition and reproduction of plant (Escobar et al., 2021). 40S ribosome protein can effectively enhance the auxin response of plants, which is essential to increase protein synthesis and maintain the normal shape of plants (Beltran-Pena et al., 2002; Uzair et al., 2021).

KEGG enrichment analysis showed that the up-regulated proteins were significantly enriched in Oxidative phosphorylation signaling pathways. Oxidative phosphorylation plays an important role in maintaining pollen tube development and pollen fertility, and is the basis of regulating pollen formation and development (Flores-Herrera et al., 2019; Wei et al., 2020). In addition, MBF1 and cysteine proteinase inhibitor were most significantly up-regulated during pollen maturation. MBF1 is an important transcriptional coactivator, which is expressed differently in plant tissues and is essential for gene expression and regulation in abiotic stress response (Hui et al., 2022). Overexpression of MBF1 in arabidopsis reduces its tolerance to low temperature and high salinity stress (Guo et al., 2014). Cysteine proteinase is expressed in various pollens, and the increased activity of this enzyme can significantly increase pollen allergenicity (Gunawan et al., 2008). Therefore, the change of the content of the above substances is not only conducive to pollen maturation, but also enhance allergenicity of pollen. In addition, endoglucanase and GDSL esterase were the most significantly down-regulated in the poplar flocs maturation process. However, studies have shown that endoglucanase and GDSL esterase are indispensable and highly expressed in the ripening process of various fruits and *Triticum aestivum* (Jara et al., 2019; Watkins et al., 2019; Mejia-Mendoza et al., 2022). It can be seen that the contents of endoglucanase and GDSL esterase are significantly different in different fruits’ ripening processes. In addition, up-regulated proteins are mainly involved in signaling pathways such as ribosome, biosynthesis of amino acids, glyoxylate and dicarboxylate metabolism, which are concerned with protein and energy synthesis, improving cold resistance and oxidation resistance (Zhu et al., 2019). The results of this study are consistent with those reported above.

Metabolomics showed that a large number of DMs were stably expressed at ripening process of pollen and poplar flocs, and there was a significant correlation between the metabolites. During pollen maturation, the up-regulated metabolites were mainly involved in the synthesis and metabolism of flavonoids and glutathione. Down-regulated metabolites are mainly involved in aminoacyl-tRNA biosynthesis and arginine biosynthesis. Studies show that flavonoids can protect pollen from UV damage (Hsieh and Huang, 2007), glutathione is essential for pollen germination and pollen tube elongation (Zechmann et al., 2011), tRNA synthesis is reduced in mature pollen (Bokszczanin et al., 2015). During the ripening process, up-regulated metabolites of poplar flocs mainly participate in citrate cycle, which is the core of energy synthesis and metabolism, and the accumulation of such metabolites can promote fruit ripening (Katz et al., 2011). Therefore, the changes of metabolites levels in this study provided the material basis for the maturation of pollen and poplar flocs.

Pollen is an important source of allergens. Data was retrieved from AllergenOnline, more than 72 potential common allergens were identified in pollen and poplar flocs, most of which showed great changes in their expression levels at different developmental stages. Interestingly, the highest abundance of mature pollen allergens in this study were A0A2I4GL39 and A0A2I4EGF0 (**Supplementary 1**), which were different from that reported earlier (Guo et al., 2021). Esch, R. studied ragweed pollen in the same region for 15 years and found that the content of allergen Amb a 1 differed by 10 times (Esch, 1997). This change is the result of the plant’s response to the external environment (Grote et al., 2001; Buters et al., 2015). Co-analysis showed that potential allergens were significantly correlated with metabolites, and there was significant interaction between allergen-allergen/metabolite. For example, A0A2I4GL39 expression abundance increased most significantly during pollen maturation, and benzoic acid content is positively correlated with it (r = 0.973). Studies have shown that benzoic acid and its derivatives are involved in the pollen development of *Nicotiana* as plant hormones (Zaveska Drabkova et al., 2021). Benzoic acid can also participate in the oscillatory growth of pollen tubes by regulating the Cl (-) channel (Zonia et al., 2002). Furthermore, A0A2I4GL39 is negatively correlated with the expression of A0A833WXQ5 (Small heat shock proteins, sHSP) (r = -0.999). Most notably, the sHSPs are extremely diverse and variable in plants. As a family, sHSPs have a clear role in response to abiotic stress, but attributing specific effects to individual proteins has proved challenging (Waters, 2013). This correlation and interaction can keep protein and metabolite levels relatively stable, so as to respond stably to various abiotic stresses.

Clinical evidence showed that 6.8% of hay fever patients were allergic to pollen of *P. deltoides*, and during the flaying of the poplar flocs in the air, the sensitization rate of grass pollen to the population can be increased (Kadocsa et al., 1993). Birch pollen is known to be the most important source of allergens. In this study, it was found that the amino acid sequence of the common allergen A0A2I4EH85 in the pollen and poplar floc was 82.3% similar to that of the birch allergen Bet v 3, which easily caused cross-allergic reaction. Bet v 3 is a 24KD polcalcin-like protein from *Betula verrucosa*, which can be bound by IgE in about 10% of pollen allergic patients (Seiberler et al., 1994). A0A2I4HKT5 and A0A2I4GR27 are highly consistent with the amino acid sequences of Hev b 7 and Hev b 8 from *Hevea brasiliensis* (83.1% and 98.5%), respectively. Hev b 7 is a 42KD patatin-like protein; about 45% of the healthy population is positive for the allergen skin prick test (SPT), and 68% of the IgE in the positive population can bind to this allergen (Bernstein et al., 2003). Hev b 8 is a 15KD profilin protein to which IgE can bind in about 39% of patients with latex allergy (Nieto et al., 2002). Cor a 2 is an important allergen from *Corylus avellana*, 15.4% of 65 hazelnut allergic patients had IgE biding to it; the common allergen A0A2I4HKI3 is highly similar to the amino acid sequence of Cor a 2 (98.5%) (Lauer et al., 2004). It is worth noting that the common allergens are highly homologous to the allergens derived from mites, and are prone to cross-allergic reactions, such as Der p 10/28/33 and Tyr p28. In addition, WB confirmed the same immunogenicity and immunoreactivity between pollen and poplar flocs, and the most important common allergens in the two groups were at about 17KD, which supported the proteomic identification.

In summary, this study combined proteomics and metabolomics to conduct a preliminary study on the ripening mechanism about pollen and poplar flocs of *P. deltoides*. It is described that there are common allergen components and their expression characteristics during maturation. Finally, WB confirmed the same biological activity between the two groups, which is equivalent to prolong the exposure time of allergens. Allergic diseases may occur in late flowering and poplar flocs period. In conclusion, this study adds to our understanding of the molecular processes of pollen and poplar flocs maturation in *P. deltoides*, and confirms the existence of common allergens between the two groups, providing a theoretical support for the prevention and treatment of allergic diseases.

## Data availability statement

The **Supplementary Material** presented in the study are deposited in the Figshare repository, accession number https://figshare.com/search?q=10.6084%2Fm9.figshare.23258639.

## Ethics statement

The animal study was reviewed and approved by The Institutional Animal Care and Use Committee of Hangzhou Hibio Technology Co. Ltd.

## Author contributions

WG and YX conceived the idea and wrote the manuscript. HLuo, YC, ZJ, and HLiu contributed for sample collection and analyzed data. JZ and CS contributed for animal experiment. YX contributed for overall editing and supervision. All authors contributed to the article and approved the submitted version.
